# Loss of Function of Fatty Acid Desaturase 7 in Tomato Enhances Photosynthetic Carbon Fixation Efficiency

**DOI:** 10.3389/fpls.2020.00932

**Published:** 2020-06-26

**Authors:** Janithri S. Wickramanayake, Josue A. Goss, Min Zou, Fiona L. Goggin

**Affiliations:** ^1^ Department of Entomology and Plant Pathology, University of Arkansas, Fayetteville, AR, United States; ^2^ Department of Mechanical Engineering, University of Arkansas, Fayetteville, AR, United States

**Keywords:** omega-3 fatty acid desaturase, FAD7, *spr2*, *acx1*, *Solanum lycopersicum*, gas exchange, chlorophyll *a* fluorescence, photosynthesis

## Abstract

Fatty Acid Desaturase 7 (FAD7) generates polyunsaturated fatty acids, promoting the desaturation of chloroplast membranes; it also provides an essential precursor for the synthesis of jasmonic acid (JA), a phytohormone that can influence plant growth, development, and primary metabolism. This study examined the effects of *spr2*, a null mutation in *SlFAD7*, on the growth, morphology, and photosynthetic traits of tomato, *Solanum lycopersicum*. Although the *spr2* mutant had a lower density of stomata than wild type plants, the two genotypes had comparable stomatal conductance, transpiration rates, and intracellular CO_2_ levels; in addition, *spr2* had significantly thinner leaf blades, which may help maintain normal levels of CO_2_ diffusion despite the lower number of stomata. Surprisingly, *spr2* also had significantly higher carbon assimilation (A) and maximum quantum efficiency of PSII (F_v_/F_m_) than wild type plants at both of the light intensities tested here (220 or 440 µmol m^−2^ s^−1^), despite having lower levels of chlorophyll than wild type plants under low light (220 µmol m^−2^ s^−1^). Furthermore, CO_2_ response curves indicated higher *in vivo* Rubisco activity (V_cmax_) in *spr2* compared to wild type plants, as well as an enhanced maximum rate of electron transport used in the regeneration of ribulose-1,5-bisphosphate (J_max_). These data indicate that loss of function of FAD7 can enhance the efficiency of both light-dependent and light-independent reactions in photosynthesis. Consistent with this, the *spr2* mutant also displayed enhanced growth, with significantly more leaves and a more compact growth habit. In contrast to *spr2*, another tomato mutant impaired in JA synthesis (*acx1*) showed no enhancements in growth or photosynthetic efficiency, suggesting that the enhancements observed in *spr2* are independent of the effects of this mutation on JA synthesis. These data demonstrate that loss of function of FAD7 can enhance photosynthesis and growth, potentially through its impacts on the chloroplast membranes.

## Introduction

Fatty Acid Desaturase 7 (FAD7) is an ω-3-desaturase found throughout the plant kingdom that influences the physical properties of chloroplast membranes. It is a membrane-associated protein located in the chloroplast, and it converts dienoic fatty acids such as linoleic acid (C18:2) to trienoic fatty acids such as linolenic acid (C18:3) (Browse et al., 1986; Iba et al., 1993). Levels of FAD7 activity modulate the relative abundance of trienoic versus dienoic fatty acids that are in membrane lipids. This ratio influences the physical characteristics of the lipids, such as their tendency to form a lipid bilayer and the temperature at which they transition from a solid (gel) to a liquid phase within that bilayer (Gounaris et al., 1983; Quinn and Williams, 1983). These characteristics in turn impact membrane structure and function; for example, high levels of desaturation lower the melting temperature of lipids and increase membrane fluidity, promoting the lateral diffusion of lipids, proteins, and other molecules within the membrane (Murata and Los, 1997). Through its effects on membrane properties, FAD7 activity could potentially influence photosynthesis, which is dependent upon membrane-associated protein complexes in the chloroplast.

FAD7 also enables the synthesis of jasmonic acid (JA), a phytohormone that regulates plant growth and defense (Schaller et al., 2004). Loss of function of FAD7 reduces JA accumulation as a result of decreased levels of C18:3, the precursor for JA synthesis. In tomato, the *spr2* mutation in *SlFAD7* results in >90% reductions in foliar C18:3 levels and wound-inducible JA accumulation (Li et al., 2002; Li et al., 2003). In Arabidopsis, loss of function of *AtFAD7* has a less pronounced effect on C18:3 levels (~60% reduction compared to wild type plants) (Browse et al., 1986), and strong inhibition of JA synthesis requires a triple mutant (*fad3fad7fad8*) that is also impaired in other ω-3-desaturases in the chloroplast (FAD8) and endoplasmic reticulum (FAD3) (McConn and Browse, 1996). Thus, the extent to which loss of function of FAD7 impacts fatty acid profiles and jasmonate signaling varies among plant species depending upon the level of functional redundancy among different ω-3-desaturases.

Because of its impacts on membrane properties and defense signaling, FAD7 can modulate resistance to multiple biotic and abiotic stresses. Overexpression of *FAD7* in tobacco enhances tolerance of low temperatures (Khodakovskaya et al., 2006; Domínguez et al., 2010), and antisense suppression of this gene increases susceptibility to salinity and drought (Im et al., 2002), indicating that *FAD7* contributes to plant adaptation to chilling, salt stress, and water limitation. On the other hand, transgenic silencing of *FAD7* in tobacco enhances tolerance of short-term heat stress (Murakami et al., 2000); this suggests that decreased ω-3-desaturase activity is adaptive under high temperatures, although complete loss of ω-3-desaturase activity compromises long-term adaptation to heat stress (Routaboul et al., 2012). These impacts on abiotic stress tolerance are thought to be due to the influence of FAD7 on membrane fluidity and stability (Upchurch, 2008).

FAD7 also influences biotic stress resistance. Mutagenesis of *FAD7* compromises JA-dependent defenses against caterpillars in tomato (Li et al., 2002), and silencing this gene in tobacco renders plants more susceptible to bacterial, viral, and oomycete infection (Im et al., 2004). On the other hand, null mutations in *FAD7* enhance aphid resistance in tomato and Arabidopsis (Avila et al., 2012), and silencing of *FAD7* in rice enhances resistance to the rice blast fungus (Yara et al., 2007). Although the mechanisms of this enhanced pest resistance are not yet fully understood, they involve altered defense signaling (Yara et al., 2007; Avila et al., 2012). Together, these studies indicate that FAD7 has contrasting effects on different stress responses, helping defend against some stresses but increasing susceptibility to others. Consistent with this, levels of *FAD7* gene expression are responsive to changing conditions, including temperature, wounding, and other stresses (Nishiuchi et al., 1997; Yaeno et al., 2006; Shi et al., 2011).

Given that levels of *FAD7* expression in plants are naturally dynamic, and manipulation of *FAD7* expression is a potential approach to engineer stress resistance, it is important to characterize the effects of this gene on plant productivity. Potentially, *FAD7* could impact photosynthetic activity through its influence on the membrane composition of the chloroplast. Alternatively, *FAD7* could influence primary metabolism by enabling the synthesis of JA, which can downregulate photosynthetic activity and growth during stress-induced defense responses (Attaran et al., 2014; Huang et al., 2017; Züst and Agrawal, 2017). Several studies indicate that FAD7 influences plants’ ability to maintain photosynthetic efficiency when exposed to certain stresses. For example, under heat stress (42°C), virus induced gene silencing of *FAD7* enhances the photosynthetic efficiency of silenced tobacco plants relative to unsilenced controls, whereas overexpression of *FAD7* in tobacco helps preserve thylakoid membrane structure under cold stress (4°C) (Khodakovskaya et al., 2006; Hiremath et al., 2017). In tomato, the negative effects of cadmium exposure on chlorophyll content, photosynthetic rates, and maximum efficiency of PSII are more pronounced in the *FAD7*-impaired *spr2* mutant than in wild type plants, suggesting that *FAD7* contributes to adaptation to cadmium in wild type plants (Zhao et al., 2016). Far fewer studies have focused on the effects of modified FAD7 activity on plant health and productivity under standard growth conditions, in the absence of artificially applied stresses. Early work on the Arabidopsis *fad7-1* mutant (JB101) established that it had 15% lower chlorophyll content, a 45% reduction in the cross-sectional area of chloroplasts, and a 44% increase in the number of chloroplasts compared to wild type plants (McCourt et al., 1987); this study also reported that *fad7-1* had somewhat higher growth rates than wild type controls at temperatures above 20°C, although it was not determined whether these numerical differences were statistically significant. In contrast, a subsequent study reported that there were no differences in growth rates or photosynthetic quantum efficiency between the *fad3-2 fad7-2 fad8* triple mutant and wild type plants (McConn and Browse, 1996). These data suggest that *FAD7* can influence chloroplast morphogenesis, but that these morphological effects do not necessarily predict an impact on photosynthesis or growth. The effects of mutations in *FAD7* on plant growth relative to wild type controls could conceivably also vary with growth conditions such as light intensity, since light has been reported to influence the expression levels of certain desaturases (Kis et al., 1998). Thus, further work is needed to understand the effects of FAD7 on chloroplast function, particularly in species such as tomato in which this enzyme has a strong, dominant effect on foliar fatty acid profiles (Li et al., 2003).

The objective of this study was to determine whether FAD7 affects photosynthesis and growth in tomato (*Solanum lycopersicum*) under standard experimental growth conditions, in the absence of artificial stress treatments. Photosynthesis was assessed by measuring a variety of parameters including total chlorophyll content, gas exchange, maximum quantum efficiency of PSII (F_v_/F_m_, a measure of plants’ capacity for photosynthetic electron transport), and the efficiency of Rubisco, which catalyzes the first step of the light-independent reactions of photosynthesis. The abundance of stomata on leaf surfaces was also examined because this morphological trait influences gas exchange, and is impacted by JA signaling (Han et al., 2018). These parameters, as well as plant height and leaf number, were compared between near-isogenic wild type tomato plants and the *suppressor of prosystemin-mediated response2* (*spr2*) mutant, which carries a point mutation that abrogates function of FAD7 by introducing a premature stop codon in *SlFAD7* (Li et al., 2003). Chlorophyll content, stomatal densities, plant growth, and chlorophyll *a* fluorescence parameters were also examined in the *acyl-CoA oxidase1* (*acx1*) mutant, which blocks the β-oxidation phase of JA synthesis (Li et al., 2005). Because this mutation disrupts JA synthesis without perturbing fatty acid metabolism, comparisons with *spr2* could help assess whether any effects of *spr2* on plant physiology were mediated through its impacts on JA, or through other effects of altered fatty acid profiles.

Surprisingly, under the environmental conditions tested here, loss of function of FAD7-enhanced carbon assimilation and photosynthetic efficiency even though it also reduced chlorophyll content and stomatal abundance. No photosynthetic enhancements were observed in *acx1*, suggesting that the effects of *spr2* may be independent of its effects of JA. Our data indicate that levels of unsaturation in chloroplast membrane lipids influence the efficiency of both light-dependent and light-independent reactions of photosynthesis. These findings are novel and potentially important because relatively few mutations are known to enhance rather than inhibit photosynthesis (Tol et al., 2017).

## Materials and Methods

### Plant Material and Growth Conditions

This study utilized *suppressor of prosystemin-mediated response2* (*spr2*), the only *fad7* null mutant available in tomato (Li et al., 2003), as well as *acyl-CoA oxidase1* (*acx1*), another well-characterized mutant in tomato that strongly blocks JA synthesis (Howe, 2004; Li et al., 2005). Seeds for these genotypes were originally obtained from Dr. Gregg Howe (Michigan State University). Plants were grown in LC1 Sunshine Professional Growing Mix (Sungro Horticulture, Agawam, MA) in 3.78 L round pots, amended with slow-release fertilizer (Osmocote Plus; 15-9-12) (Scotts-MiracleGro Company, Marysville, OH), and watered with a nutrient solution containing 1000 ppm CaNO_3_ (Hydro Agri North America, Tampa, FL), 500 ppm MgSO_4_ (Giles Chemical, Waynesville, NC) and 500 ppm 4-18-38 Gromore fertilizer (Grow More, Gardena, CA). All experiments were conducted in Conviron growth chambers (temperature, 22–23°C; RH, 50–65%; photoperiod, 16 h/8 h light/dark). Unless otherwise specified, plants were grown at ~220 µmol m^−2^ s^−1^ light intensity, which was measured at the level of the top of the plant canopy using a quantum meter (MQ-200, Apogee, Instruments, Logan, UT, USA).

### Observations of Plant Growth and Morphology

Observations were made four weeks after germination (10–12 replicates plants per genotype). Plant heights were measured manually from the soil level to the apical meristem, and the number of leaves per plant was counted. Compactness was calculated by dividing the number of leaves by the plant height. Leaf thickness was measured using a Mitutoyo 500 series digital caliper (Mitutoyo America Corporation, USA) by measuring thickness at five different points on the fifth leaf below the apical meristem and averaging these values per plant. Images of stomata were captured on both the abaxial and adaxial surfaces of the fifth leaf of four-week old plants under 20× magnification using a 3D laser scanning confocal microscope (VK-X260K, Keyence Corporation, USA). The imaging Z-range was adjusted for each sample in order to capture all the stomata in the field of view (712×534 µm^2^) that was being measured. Stomata were manually marked in the digital images, and then counted using MultiFileAnalyzer software (Keyence Corporation, USA). For each biological replicate, three separate images were captured from each leaf surface (abaxial and adaxial), and the numbers of stomata in these subsamples were averaged before analysis.

### Measurement of Chlorophyll Content and Photosynthetic Parameters

Two approaches were taken to the measurement of photosynthetic parameters in this study. Initial experiments with all three genotypes (wild type, *spr2*, and *acx1*) were conducted with a handheld spectrometer—PhotosynQ MultispeQ v1.0 (Michigan, USA) (Kuhlgert et al., 2016)—that allowed the rapid estimation of chlorophyll content and chlorophyll *a* fluorescence (10–12 replicates per genotype). Then, to further characterize differences in photosynthesis observed in *spr2*, follow-up experiments on wild type and *spr2* were conducted with a photosynthesis system that enables the measurement of gas exchange in parallel with chlorophyll fluorescence (LI-6400XT, LI-COR Biosciences, Lincoln, NE, USA). For all experiments, photosynthetic measurements were taken four weeks after germination on the fifth leaf below the apical meristem, which was fully expanded and mature. Experiments using the MultispeQ and the LI-6400XT were performed in a darkened growth chamber after an overnight dark exposure (approximately 7 h) to acclimate the plants to darkness.

For experiments that used the LI-6400XT, gas exchange and fluorescence measurements were performed simultaneously using a leaf chamber fluorometer, an LED-based fluorescence attachment (LCF 6400-40, LI-COR Biosciences, Lincoln, NE, USA). During the measurements, the conditions in the LCF chamber were maintained at 400 ppm reference CO_2_ concentration, 55% to 65% relative humidity, and 300 µmol m^−2^ s^−1^ airflow. The LI-6400XT, unlike the MultiSpeQ, does not measure chlorophyll content in parallel with chlorophyll fluorescence; therefore, immediately after LI-6400XT measurements were taken, chlorophyll content was measured in the same leaves using a SPAD-502 meter (Konica Minolta Sensing, Inc., Japan) according to the manufacturer’s instructions. Because of the time required for LI-6400XT measurements, LI-COR experiments were replicated in time; for each experiment, eight plants (two plants per treatment group) were measured per day over the course of eight days to yield 16 replicates per treatment group. Planting dates for the experiment were staggered to ensure that all replicates were the same age at the time of measurement (four weeks after germination). The following parameters were analyzed using the gas exchange and fluorescence data: carbon assimilation (A); minimal fluorescence in dark-adapted leaves (F_o_, measured in the dark) maximal fluorescence in dark-adapted leaves (F_m_, measured during a rapid exposure of the leaf to a saturating light pulse); and maximum quantum efficiency of PSII (F_v_/F_m_), which is calculated as (F_m_-F_o_)/F_m_.

### Comparison of Chlorophyll Content and Photosynthesis at Different Light Intensities

The potential impacts of light intensity on chlorophyll content and photosynthesis was measured in *spr2* compared to wild type plants because previous pilot experiments had suggested that wild type plants were greener than *spr2* when plants were grown under typical growth chamber conditions (220 µmol m^−2^ s^−1^ light intensity), but not when grown in the greenhouse, which has variable but typically higher light intensities. To address this question under controlled environmental conditions, all plants were initially grown in a growth chamber at 220 µmol m^−2^ s^−1^, and then 48 h before measurements were taken, the light canopy was adjusted to transfer half of the plants to 440 µmol m^−2^ s^−1^, a moderate light intensity comparable to levels found in the greenhouse. The remaining plants were kept at 220 µmol m^−2^ s^−1^. Gas exchange and fluorescence measurements were taken four weeks after germination with a LI-6400XT, and chlorophyll content was measured with a SPAD-502 meter.

### CO_2_ Response Curves

The responses of wild type and *spr2* plants to varying intercellular CO_2_ concentrations was assessed through CO_2_ response (A/Ci) curves, which are generated by measuring the net photosynthetic rates of leaves exposed to a range of CO_2_ concentrations under saturating light conditions (1200 µmol m^−2^ s^−1^) (Tanaka et al., 2013). Plants were grown in a growth chamber at 440 µmol m^−2^ s^−1^ because of the higher photosynthetic rates observed at this light intensity. Before recording measurements, the designated leaf was placed inside the LCF chamber head, and the CO_2_ assimilation rate was allowed to stabilize at 1200 µmol m^−2^ s^−1^ light intensity, 400 ppm (ambient) CO_2_ concentration and 55% to 65% relative humidity. Once the photosynthesis rates stabilized (assessed by observing real-time graphs), the A/Ci curve was generated using the auto-program feature of the LI-6400XT to progress the CO_2_ concentration through the following sequence of levels: 300, 200, 100, 50, 400, 400, 600, 800, 1000 ppm. At each CO_2_ concentration, carbon assimilation readings were automatically recorded immediately after stabilization, which was generally within 2 min. Two 400 ppm points were logged in order to allow the leaf to recover after being exposed to low CO_2_ concentrations. The maximum carboxylation rate of Rubisco/*in vivo* apparent Rubisco activity (V_cmax_) and the maximum rate of electron transport used in the regeneration of ribulose-1,5-bisphosphate (RuBP) (J_max_) of four-week old *spr2* and wild type plants were estimated by fitting the Farquhar, von Caemmerer, Berry (FvCB) model (Farquhar et al., 1980) using the “Plantecophys” package in R software (Duursma, 2015; R Core Team, 2017). All the CO_2_ response curves were generated between 8:00 am and 12:00 pm, with six biological replicates per genotype.

### Statistical Analyses

Experiments with one variable (genotype) were analyzed by one-way ANOVA, and experiments with two variables (genotype and light intensity) in a full factorial design were analyzed by two-way ANOVA. For experiments that were replicated in time, the days on which each plant was measured (measurement days) were treated as random blocks. If significant interactions between treatments were found, mean separations were done by a Tukey’s HSD. The statistical analyses were conducted with JMP^®^ Pro 14 (SAS Institute Inc.).

## Results

### The *spr2* Mutation Decreases Chlorophyll Content and Stomatal Densities but Promotes Plant Growth, Compactness, and Enhanced Maximum Quantum Efficiency of PSII

**Figure 1:**
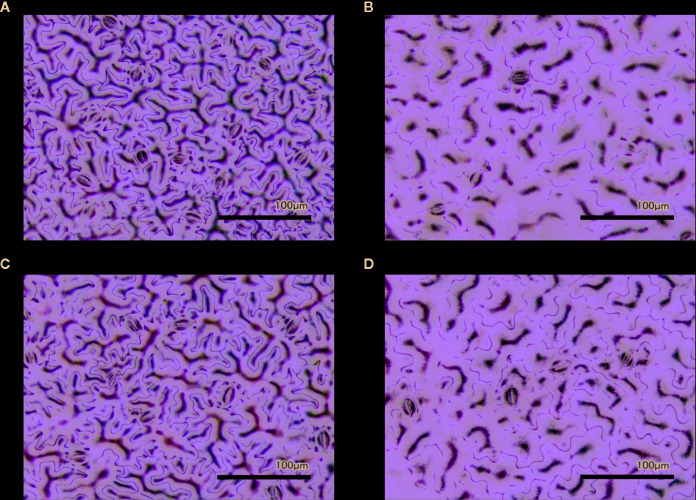
Laser-scanning micrographs of stomata. Micrographs were captured using a Keyence VK-X260K on the abaxial **(A)** and adaxial **(B)** leaf surfaces of WT plants and the abaxial **(C)** and adaxial **(D)** leaf surfaces of *spr2* plants under 20× magnification (Scale bars = 100 µm). Plants were four-weeks old and the fifth leaf was used for imaging.

**Figure 2:**
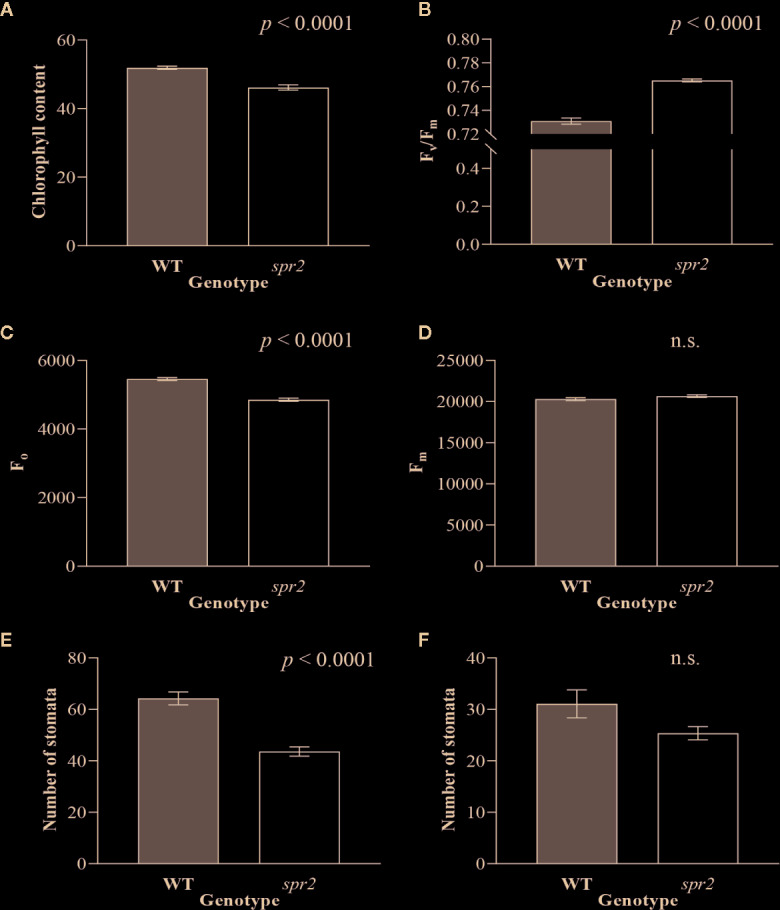
Chlorophyll content, chlorophyll fluorescence and abundance of stomata of *spr2* compared to wild type (WT) plants. Chlorophyll content **(A)**, maximum quantum efficiency of photosystem II (PSII) when dark-adapted (F_v_/F_m_) **(B)**, minimal fluorescence of dark-adapted leaves (F_o_) **(C)** and maximum fluorescence in dark-adapted leaves (F_m_) **(D)** were compared in four-week old *spr2* and WT plants using a MultispeQ instrument. The number of stomata were counted in the laser scanning micrographs captured from the abaxial **(E)** and the adaxial **(F)** leaf surfaces of WT and *spr2* using a Keyence VK-X260K under 20× magnification in the field of view (712×534 µm^2^). Error bars represent SEM; N = 10. Data were analyzed by one-way ANOVA; n.s., not significant.

In order to investigate the impacts of the *spr2* mutation on plant development and photosynthetic efficiency, plant growth measurements in *spr2* and wild type plants were taken at four weeks after germination under typical growth chamber conditions (220 µmol m^−2^ s^−1^), and chlorophyll content and the maximum quantum efficiency of PSII in dark-adapted leaves (F_v_/F_m_) were measured with a MultispeQ handheld spectrometer. To determine if the *spr2* mutation was associated with any differences in leaf morphology, stomatal densities were measured on the lower and upper leaf surfaces using 3D laser scanning confocal microscopy (**Figure 1**). Chlorophyll content in the *spr2* mutant was significantly lower than in wild type foliage by ~13% (**Figure 2A**). Despite this, F_v_/F_m_ was significantly higher in *spr2* than in WT (**Figure 2B**), indicating that the light-dependent reactions of photosynthesis are more efficient in *spr2* mutants than in wild type. F_v_/F_m_ was calculated using measurements of the minimal chlorophyll fluorescence (F_o_; **Figure 2C**) and maximum chlorophyll fluorescence (F_m_; **Figure 2D**) of dark-adapted leaves, and the difference in F_v_/F_m_ between genotypes was due to significantly lower F_o_ in *spr2* compared to wild type controls. The enhanced photosynthetic efficiency of *spr2* was not associated with more abundant stomata for gas exchange; in fact, compared to wild type plants, the *spr2* mutant had 32% fewer stomata on the lower (abaxial) leaf surface, where the majority of stomata are located (**Figure 2E**). The number of stomata on the upper (adaxial) surface did not differ between *spr*2 and wild type plants (**Figure 2F**). Mutant plants also had significantly thinner leaf blades (**Table 1**). Consistent with enhanced photosynthetic efficiency, the *spr2* mutant showed enhanced growth; although the mutants were similar in height to wild type plants, they had significantly more leaves (a 27% increase), reflecting a more compact growth habit (**Table 1**). These data indicate that the *spr2* mutation promotes growth and photosynthetic efficiency even while limiting chlorophyll content and stomatal development.

**Table 1:** Growth and morphology of *spr2* compared to wild type (WT) plants.

	WT	*spr2*	*p*-value
**Height (cm)**	22.3 ± 0.8	21.9 ± 1.4	0.4
**Number of leaves**	8.1 ± 0.7	10.3 ± 0.7	<0.0001
**Compactness**	0.36 ± 0.04	0.47 ± 0.03	<0.0001
**Leaf thickness (mm)**	0.37 ± 0.009	0.32 ± 0.009	0.003

Plants were phenotyped at 4 weeks after germination. Plant height was measured manually and the leaf thickness was measured using a Mitutoyo series 500 digital caliper. The values reported are ± SEM; N = 10. Data were analyzed by one-way ANOVA.

### The *acx1* Mutation Also Suppresses Chlorophyll Content but Does Not Significantly Impact Plant Growth, Morphology, or Photosynthetic Efficiency

**Figure 3:**
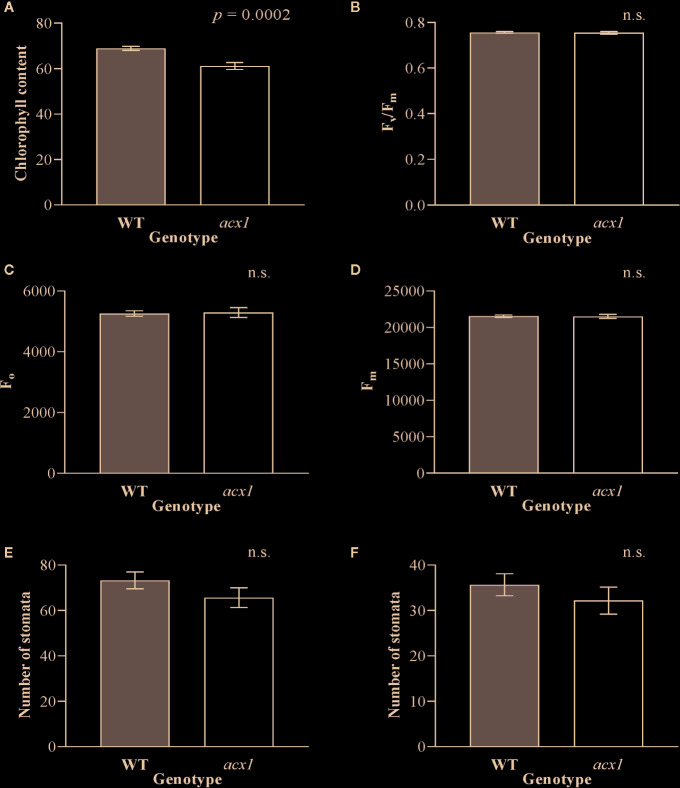
Chlorophyll content, chlorophyll fluorescence and abundance of stomata of *acx1* compared to wild type (WT) plants. Chlorophyll content **(A)**, maximum quantum efficiency of photosystem II (PSII) when dark-adapted (F_v_/F_m_) **(B)**, minimal fluorescence of dark-adapted leaves (F_o_) **(C)** and maximum fluorescence in dark-adapted leaves (F_m_) **(D)** were compared in four-week old *acx1* and WT plants using a MultispeQ instrument; N = 12 for WT and N = 10 for *acx1*. The number of stomata were counted in the laser scanning micrographs captured from the abaxial **(E)** and the adaxial **(F)** leaf surfaces of WT and *acx1* using a Keyence VK-X260K under 20× magnification in the field of view (712 × 534 µm^2^); N = 10. Data were analyzed by one-way ANOVA; n.s., not significant. Error bars represent SEM.

In order to explore whether the impact of *spr2* on growth and photosynthesis could be due to reduction of JA synthesis, we also measured plant growth, morphology, chlorophyll content, and chlorophyll fluorescence in another tomato mutant line with impaired JA synthesis, *acx1*. Similar to *spr2*, *acx1* had significantly lower chlorophyll levels than wild type plants (**Figure 3A**). However, in contrast to *spr2*, the *acx1* mutation did not impact plant growth (**Table 2**), chlorophyll fluorescence parameters (**Figures 3B–D**), or stomatal densities (**Figures 3E, F**). These results suggest that altered JA levels may play a role in the decreased chlorophyll content of the *spr2* mutant, but that the beneficial effects of *spr2* on plant growth, morphology, or photosynthetic efficiency may be independent of its effects on JA synthesis.

**Table 2:** Growth and morphology of *acx1* compared to wild type (WT) plants.

	WT	*acx1*	*p*-value
**Height (cm)**	24.4 ± 2.4	24.1 ± 1.6	0.7
**Number of leaves**	9.8 ± 1.3	9.5 ± 1.7	0.6
**Compactness**	0.40 ± 0.05	0.39 ± 0.08	0.8
**Leaf thickness (mm)**	0.33 ± 0.01	0.34 ± 0.03	0.9

Plants were phenotyped at 4 weeks after germination. Plant height was measured manually and the leaf thickness was measured using a Mitutoyo series 500 digital caliper. The values reported are ± SEM; N = 12 for WT and N = 10 for acx1. Data were analyzed by one-way ANOVA.

### The Effects of *spr2* on Chlorophyll Content Vary With Light Intensity

**Figure 4:**
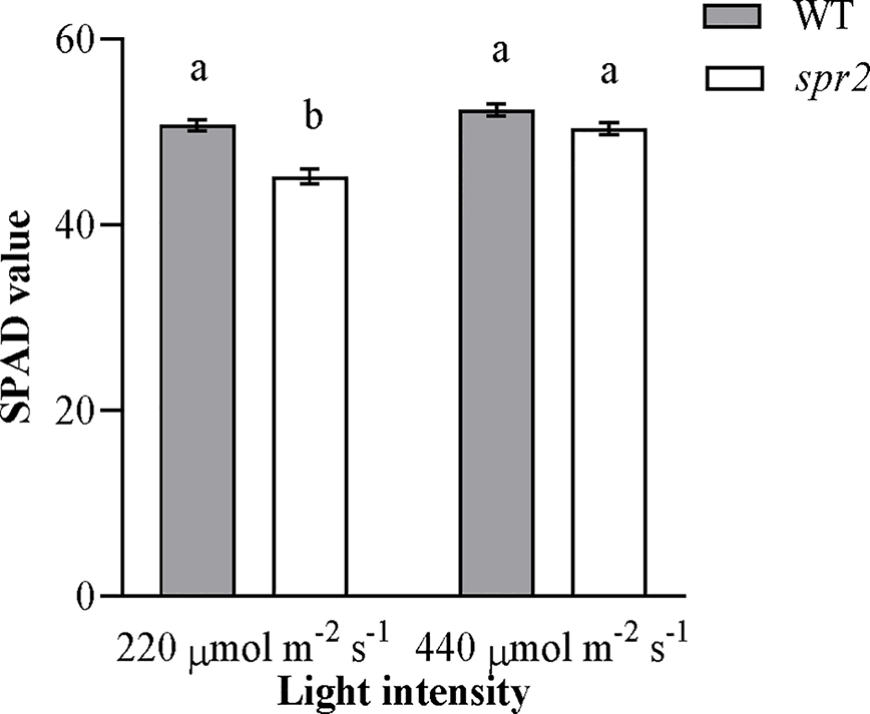
Chlorophyll content of *spr2* and wild type (WT) plants at two light intensities. Chlorophyll content was measured in four-week old plants using a SPAD meter. Error bars represent SEM; N = 16. According to a two-way ANOVA, there was a significant interaction between genotype and light level (P = 0.015), and so mean separations were performed with Tukey-Kramer HSD. Bars labeled with different letters differ significantly at α = 0.05.

Because preliminary observations suggested that chlorophyll abundance in *spr2* might vary with light intensity, chlorophyll levels were compared using a SPAD meter in plants grown under standard growth chamber conditions (220 µmol m^−2^ s^−1^ light exposure) and plants transferred to a higher light intensity (440 µmol m^−2^ s^−1^) 48 h before measurement. Both genotype and light intensity had a significant effect on chlorophyll content, and the effects of genotype varied depending upon the light intensity (two-way ANOVA: Genotype: *p =* 0.0004; Light intensity: *p*<0.0001; Genotype x Light intensity: *p =* 0.015). At 220 µmol m^−2^ s^−1^ light, chlorophyll levels were significantly lower in *spr2* than in wild type plants (**Figure 4**), similar to our previous observations (**Figure 2A**). However, at 440 µmol m^−2^ s^−1^, chlorophyll content of *spr2* increased by more than 10% compared to the levels observed at 220 µmol m^−2^ s^−1^, and was statistically comparable to levels in wild type plants (**Figure 4**). These data indicate that while *spr2* inhibits chlorophyll accumulation under low light, this effect can be diminished by exposure to higher light levels.

### The *spr2* Mutation Enhances Carbon Assimilation and Maximum Quantum Efficiency of PSII Independent of Its Effects on Chlorophyll Content

**Figure 5:**
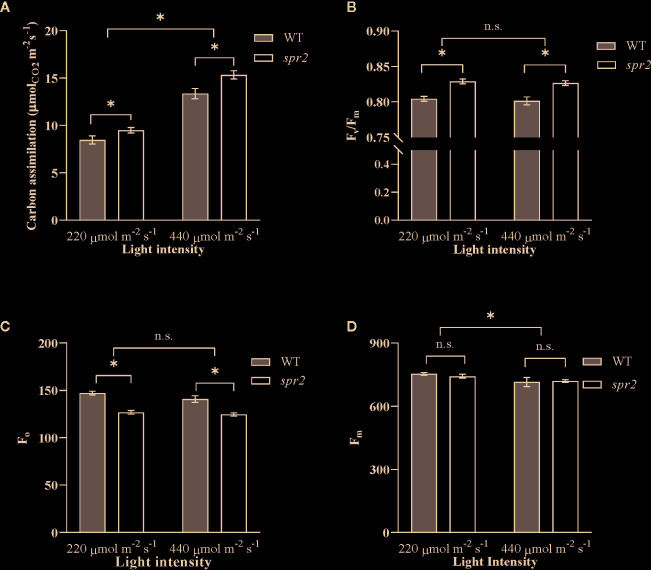
Carbon assimilation and chlorophyll fluorescence of *spr2* compared to wild type (WT) plants. Carbon assimilation **(A)**, maximum quantum efficiency of photosystem II (PSII) when dark-adapted (F_v_/F_m_) **(B)**, minimal fluorescence of dark-adapted leaves (F_o_) **(C)** and maximum fluorescence in dark-adapted leaves (F_m_) **(D)** were compared in four week old *spr2* and WT plants using a LI-6400XT. Error bars represent SEM; N = 16. Pairwise comparisons marked with asterisks denote significant main effects according to two-way ANOVA at α = 0.05, and n.s., not significant. No significant interactions were observed between main effects (P > 0.05).

To further characterize the effects of *spr2* on photosynthesis, and to determine whether these effects vary with light intensity, photosynthetic activity was also compared in *spr2* and WT plants at light intensities of 220 and 440 µmol m^−2^ s^−1^ using a portable photosynthesis system capable of measuring gas exchange as well as chlorophyll fluorescence (LICOR LI-6400XT). Carbon assimilation (A), a measure of overall photosynthetic activity, increased with increasing light intensity, and was significantly higher by 12% to 15% in *spr2* than in wild type plants (**Figure 5A**). There was no significant interaction between genotype and light intensity, suggesting that the effects of *spr2* on carbon assimilation were similar at the two light levels tested here (Two-way ANOVA: Genotype: *p =* 0.0038; Light intensity: *p*<0.0001; Genotype x Light intensity: *p* = 0.18). Stomatal conductance, transpiration rates, and intracellular CO_2_ levels were comparable between genotypes at both light levels (**Table 3**), indicating that the lower stomatal densities observed in *spr2* are not a limitation on gas exchange.

**Table 3:** Gas exchange parameters of *spr2* and wild type (WT) tomato plants.

	220 µmol m^−2^ s^−1^		440 µmol m^−2^ s^−1^		*p*-value		
	WT	*spr2*	WT	*spr2*	Genotype	Light Intensity	Interaction
**Stomatal conductance**	0.14 ± 0.058	0.18 ± 0.055	0.19 ± 0.067	0.22 ± 0.065	0.10	0.003	0.78
**Intercellular CO_2_**	277.86 ± 31.22	290.45 ± 21.67	258.84 ± 27.98	255.69 ± 30.24	0.50	<0.0001	0.13
**Transpiration**	2.00 ± 0.69	2.36 ± 0.51	2.70 ± 0.71	3.01 ± 0.69	0.09	0.0002	0.87

Plants were measured at 4 weeks after germination using a LI-6400XT. The values reported are ± SEM; N = 16. Data were analyzed by two-way ANOVA.

Chlorophyll fluorescence measurements from the LICOR LI-6400XT also confirmed enhanced maximum quantum efficiency of PSII in *spr2* due to decreased F_o_. F_v_/F_m_ was significantly higher in *spr2* than in wild type plants, and was not significantly impacted by light intensity (Two-way ANOVA: Genotype: *p*<0.0001; Light intensity: *p =* 0.45; Genotype x Light intensity: *p =* 0.92) (**Figure 5B**). Similar to our previous observations with the MultispeQ, F_o_ was significantly lower in *spr2* than in wild type plants (**Figure 5C**) (Two-way ANOVA: Genotype: *p =* 0.0002; Light intensity: *p =* 0.062; Genotype x Light intensity: *p =* 0.35), and F_m_ did not vary significantly between genotypes or light intensities (**Figure 5D**) (Two-way ANOVA: Genotype: *p =* 0.63; Light intensity: *p =* 0.008; Genotype x Light intensity: *p =* 0.44). These results support the hypothesis that the light-dependent reactions of photosynthesis are more efficient in *spr2* mutants than in wild type. They also suggest that the effects of *spr2* on carbon assimilation and PSII efficiency are independent of its effects on chlorophyll content, because, unlike changes in chlorophyll content, they are observed at both light intensities.

### The *spr2* Mutant Has Higher Rubisco Carboxylase Activity and Maximum Rates of Photosynthetic Electron Transport Than Wild Type Plants

**Figure 6:**
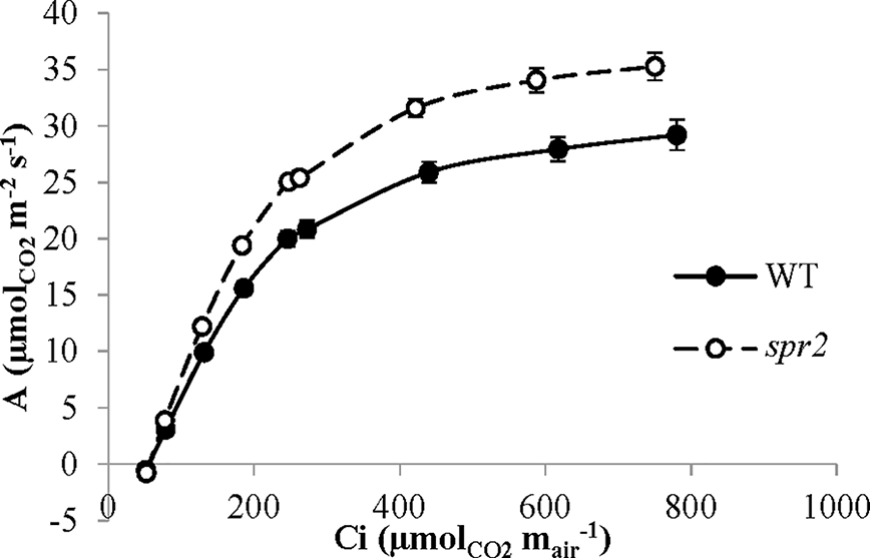
CO_2_ response (A/Ci) curves of *spr2* and wild type (WT) tomato plants. Carbon assimilation (A) was measured at multiple CO_2_ concentrations in four week old plants with a LI-6400XT, and A was graphed against CO_2_ concentration (Ci). Error bars represent SEM; N = 6.

CO_2_ response curves (A/Ci curves) were also observed in *spr2* and WT plants to estimate the Rubisco-mediated carboxylation rate (V_cmax_) and the maximum rate of electron transport used in the regeneration of RuBP (J_max_). The CO_2_ response curves generated are shown in **Figure 6**, and revealed that both V_cmax_ and J_max_ were significantly higher in *spr2*, exceeding the rates observed in wild type plants by more than 20% (**Table 4**). These results indicate that Rubisco-mediated carboxylation and RuBP regeneration are less of a limitation on photosynthesis in *spr2* than in wild type plants, and that *spr2* has enhancements in both light-dependent (J_max_) and light-independent (V_cmax_) photosynthetic reactions. The ratio of V_cmax_ to J_max_ is also significantly higher in *spr2* than in wild type plants (**Table 4**).

**Table 4:** Maximum carboxylation rate of Rubisco (V_cmax_) and maximum rate of electron transport used in the regeneration of RuBP (J_max_) of *spr2* and wild type (WT) tomato plants.

	WT	*spr2*	*p*-value
**V_cmax_ (µmol m^−2^ s^−1^)**	119.62 ± 7.63	148.43 ± 8.58	0.03
**J_max_ (µmol m^−2^ s^−1^)**	165.99 ± 9.20	227.53 ± 14.06	0.004
**V_cmax_/J_max_**	1.39 ± 0.03	1.54 ± 0.05	0.04

V_cmax_ and J_max_ were calculated using CO_2_ response curves and are reported ± SEM; N = 6.

## Discussion

Here we demonstrate that a mutation in tomato that impairs FAD7 function (*spr2*) enhances growth and photosynthetic activity. Compared to wild type plants, the *spr2* mutant was comparable in height but had significantly more leaves, resulting in a more compact growth habit. The *fad7-1* mutant in Arabidopsis was also reported to have a slightly higher relative growth rate than wild type plants at temperatures above 20°C (McCourt et al., 1987). To explore the basis for enhanced vegetative growth in *spr2*, we measured photosynthetic activity and chlorophyll content, potential limiting factors in plant productivity. These parameters were compared at two light intensities (220 µmol m^−2^ s^−1^ and 440 µmol m^−2^ s^−1^) because our preliminary observations suggested that the foliage of the two genotypes varied in color under some but not all light regimes. At 220 µmol m^−2^ s^−1^, foliar chlorophyll levels were significantly lower in *spr2* than in wild type plants, and this is consistent with a prior report that loss of function of FAD7 in Arabidopsis also reduces chlorophyll content (McCourt et al., 1987). However, when *spr2* and wild type plants were grown under moderate light intensity (440 µmol m^−2^ s^−1^), the two genotypes had statistically comparable chlorophyll levels. Furthermore, carbon assimilation was significantly higher in *spr2* than in wild type under both light regimes, regardless of the lower chlorophyll content in *spr2* at 220 µmol m^−2^ s^−1^. Certain soybean mutants with reduced chlorophyll content can also maintain normal levels of carbon assimilation, demonstrating that chlorophyll content is not always a rate-limiting factor in photosynthesis (Walker et al., 2018). Likewise, *spr2* maintained normal stomatal conductance, transpiration rates, and intracellular CO_2_ levels despite lower than normal stomatal densities. The thinner leaf blades of *spr2* may possibly compensate for the lower density of stomata, enabling the rapid diffusion of CO_2_ in the intracellular spaces. Thus, the abundance of stomata does not necessarily impact rates of carbon assimilation; in fact, very high stomatal densities can actually decrease the efficiency of gas exchange at individual stomata (Vráblová et al., 2017). Rather than chlorophyll content or stomatal densities being rate-limiting, our data suggest that rates of photosynthetic activity in *spr2* are determined by the efficiency of electron transport and of the light-independent reactions of photosynthesis.

Enhanced photosynthetic efficiency in *spr2* was associated with enhancements in the light-dependent reactions of photosynthesis. Measurements of carbon assimilation in response to varying concentrations of carbon dioxide (A/Ci curves) indicated that J_max_ was significantly higher in *spr2* than in wild type plants. This parameter is one of the most important constraints on photosynthesis when light is not limiting (Long and Bernacchi, 2003). Measures of chlorophyll fluorescence also corroborated the enhancement of light-dependent reactions in *spr2*. The maximum quantum yield of PSII (F_v_/F_m_), a widely used measure of the efficiency of the light-dependent reactions, was significantly greater in *spr2* compared to wild type. The ratio F_v_/F_m_ is calculated based on measurements of F_m_ in response to saturating light and F_o_ in the absence of light levels adequate for photosynthesis (Baker, 2008). The increase in F_v_/F_m_ in *spr2* was due to significant reductions in F_o_, a parameter that is correlated with oxidative stress resulting from photodamage (Krause and Weis, 1991; Baker, 2008; Rodrigues et al., 2008). Together, these data indicate that the light-dependent reactions of photosynthesis are more efficient in *spr2* than in wild type plants.

In addition to promoting light-dependent photosynthetic reactions, the *spr2* mutation also enhances light-independent reactions. Carbon dioxide response curves revealed that the carboxylase activity of Rubisco (as measured by V_cmax_) was significantly greater in *spr2* than in wild type plants. This enzyme catalyzes carbon fixation, the first step in sugar synthesis by the light-independent reactions of photosynthesis (i.e. the Calvin cycle). Although Rubisco is localized in the stroma, it is found in protein complexes bound to the outer surface of the thylakoids (Suss et al., 1993), and so could be directly impacted by the lipid composition of the thylakoid membranes. Degradation or inactivation of Rubisco causes decreases in V_cmax_ (Zhou et al., 2004). As a notoriously inefficient enzyme, Rubisco represents a major rate limitation in carbon assimilation, and a frequently-proposed target for crop improvement (Parry et al., 2013). Together with J_max_ and triose-phosphate utilization, V_cmax_ is one of the three most important physiological limitations on photosynthesis (Long and Bernacchi, 2003). Even though V_cmax_ is enhanced in *spr2*, this mutant is also characterized by a significantly higher J_max_/V_cmax_ ratio compared to wild type plants. The J_max_/V_cmax_ ratio is thought to reflect a plant’s resource allocation to electron transport proteins relative to Rubisco, and these investments can vary depending upon environmental conditions (Onoda et al., 2005; Akita et al., 2012; Walker et al., 2014). For example, under light conditions that induce photodamage, more rapid turnover of the D1 reaction center protein may be required to limit photoinhibition (Andersson and Aro, 2001). Carbon dioxide concentrations in the environment may also influence the J_max_/V_cmax_ ratio; V_cmax_ is the primary rate limitation on photosynthesis at low CO_2_, whereas J_max_ is a more important limitation under high CO_2_ conditions (Onoda et al., 2005; Akita et al., 2012). The high J_max_/V_cmax_ in *spr2* therefore may help this mutant to optimize utilization of light energy when CO_2_ is abundant, and it is of special interest because it could also be a beneficial adaptation under increasing environmental CO_2_ levels (Akita et al., 2012; Walker et al., 2014; Kromdijk and Long, 2016).

The enhanced photosynthetic capacity observed in the *spr2* mutant appears to be independent of the effects of this mutation on JA signaling, because similar effects were not observed in *acx1*, another mutant impaired in JA synthesis. Although *acx1* and *spr2* both suppressed chlorophyll accumulation relative to wild type plants, only *spr2* significantly promoted growth or influenced chlorophyll fluorescence parameters such as F_v_/F_m_. Furthermore, whereas the JA-deficient *spr2* mutant had reduced stomatal abundance and a compact growth habit with short internodes, JA was previously reported to suppress stomatal development in Arabidopsis (Han et al., 2018) and internode elongation in coyote tobacco (*Nicotiana attenuata*) (Heinrich et al., 2013). Thus, the effects of *spr2* on growth and photosynthesis are more likely to be mediated through alterations in chloroplast membranes than through inhibition of JA synthesis.

Loss of function of FAD7 may impact numerous aspects of membrane structure and lipid metabolism in the chloroplast. The *spr2* mutation decreases the content of trienoic fatty acids and increases the abundance of dienoic fatty acids, particularly in lipids that are synthesized in the chloroplast such as monogalactosyldiacylglycerol (MGDG), digalactosyldiacylglycerol (DGDG), sulfoquinovosyldiacylglycerol (SQDG), and phosphatidylglycerol (PG) (Li et al., 2003). In general, decreased levels of desaturation cause denser packing of membrane lipids (i.e. higher membrane order) and decreased membrane fluidity, which could impact the functioning of photosynthetic proteins embedded in or associated with the thylakoid membranes (Popova et al., 2007). However, these membrane properties have not been measured in *spr2*, and the *fad7-1* mutant in Arabidopsis is reported to have normal levels of membrane fluidity in the absence of stress treatments (McCourt et al., 1987). Another feature of membrane structure that could be impacted by *spr2* is the relative abundance of different lipids in the chloroplast membranes, because antisense suppression of *SlFAD7* in tomato has been shown to increase the abundance of PG and decrease the ratio of MGDG to DGDG (Liu et al., 2006). PG is a cofactor for PSII that is critical for photosynthesis, and the MGDG/DGDG ratio is very important to the structure of thylakoid membranes (Boudière et al., 2014). Therefore, further investigation is warranted to determine whether changes in the chemical composition or physical structure of the thylakoid membranes contribute to enhanced photosynthesis in *spr2*. It would also be useful to measure basal and stress-responsive free fatty acid pools in *spr2* and wild type plants, because exogenous free linolenic acid inhibits electron transport at PSII and PSI (Siegenthaler, 1974; Golbeck et al., 1980), and endogenous linolenic acid has been implicated in heat-induced oxidative damage to photosynthetic proteins (Yamauchi et al., 2008). If the endogenous levels of free linolenic acid generated by lipases in wild type plants at moderate temperatures are adequate to have any inhibitory effects on photosynthesis, decreased linolenic acid content in *spr2* could contribute to the enhancements in photosynthesis we have observed in this genotype. Consistent with the lower F_o_ values observed in *spr2*, decreased linolenic acid levels could potentially also decrease lipid peroxidation and the consequent accumulation of malonaldialdehyde (MDA), which damages PSII and Rubisco (Yamauchi et al., 2008). Thus, further investigation of lipid peroxidation and reactive oxygen species (ROS) accumulation in the chloroplast is merited to explore possible mechanisms of enhanced photosynthesis in *spr2*. Observations of *spr2* over a temperature gradient could also help address whether the enhanced photosynthesis observed here under moderate temperature (~23°C) is related to or distinct from the enhanced short-term thermotolerance associated with impaired FAD7 function (Murakami et al., 2000; Routaboul et al., 2012; Hiremath et al., 2017).

This is to our knowledge the first report that modification of fatty acid desaturation can significantly enhance photosynthesis in the absence of stress treatments. It provides a foundation for future investigations into chloroplast structure and function in *spr2*, which could in turn shed light on routes to enhance photosynthetic efficiency.

## Data Availability Statement

The raw data supporting the conclusions of this article will be made available by the authors, without undue reservation.

## Author Contributions

JW performed the experiments and statistical analyses, and wrote the first draft of the manuscript. JG and MZ assisted with image acquisition of stomata and FG conceived and supervised the project, and completed the final manuscript. All authors contributed to the article and approved the submitted version.

## Funding

This research was supported by the AFRI Foundational Program [grant 2015-67013-23412 to FG] from the USDA National Institute of Food and Agriculture, as well as the Center for Advanced Surface Engineering (CASE), under the National Science Foundation (NSF) grant OIA-1457888 and the Arkansas EPSCoR Program. Support was also provided by the University of Arkansas Chancellor’s office and the Arkansas Division of Agriculture.

## Conflict of Interest

The authors declare that the research was conducted in the absence of any commercial or financial relationships that could be construed as a potential conflict of interest.

## Abbreviations

A, carbon assimilation; A/Ci curve, CO_2_ response curve; F_o_, minimal fluorescence of a dark-adapted leaf; F_m_, maximal fluorescence of a dark-adapted leaf; F_v_, variable fluorescence of a dark-adapted leaf; F_v_/F_m_, maximum photochemical efficiency of PSII of a dark-adapted leaf; J_max_, maximum rate of electron transport used in the regeneration of ribulose-1,5-bisphosphate (RuBP); RH, relative humidity; V_cmax_, maximum carboxylation rate of Rubisco/*in vivo* apparent Rubisco activity.
